# Therapeutic Hypothermia With Progesterone Improves Neurologic Outcomes in Ventricular Fibrillation Cardiac Arrest After Electric Shock

**DOI:** 10.7759/cureus.15749

**Published:** 2021-06-18

**Authors:** Fred N Qafiti, David Rubay, Rebecca Shin, Lawrence Lottenberg, Robert Borrego

**Affiliations:** 1 General Surgery, Charles E. Schmidt College of Medicine, Florida Atlantic University, Boca Raton, USA; 2 Trauma and Surgical Critical Care, University of Florida College of Medicine, Gainesville, USA; 3 Surgery, Charles E. Schmidt College of Medicine, Florida Atlantic University, Boca Raton, USA; 4 Surgery, St. Mary's Medical Center, Florida Atlantic University, West Palm Beach, USA; 5 Surgery, St. Mary's Medical Center, West Palm Beach, USA

**Keywords:** electric shock, hypothermia, out-of-hospital cardiac arrest, progesterone, anoxic brain injury

## Abstract

Trauma by electricity imposes mechanical, electrical, and thermal forces on the human body. Often, the delicate cardiac electrophysiology is disrupted causing dysrhythmia and subsequent cardiac arrest. Anoxic brain injury (ABI) is the most severe consequence and the main cause of mortality following cardiac arrest. Establishing a working protocol to treat patients who are at risk for ABI after suffering a cardiac arrest is of paramount importance. There has yet to be sufficient exploration of combination therapy of therapeutic hypothermia (TH) and progesterone as a neuroprotective strategy in patients who have suffered cardiac arrest after electric shock.

The protocol required TH initiation upon transfer to the ICU with a target core body temperature of 33°C for 18 hours. This was achieved through a combination of cooling blankets, ice packs, chilled IV fluids, nasogastric lavage with iced saline, and intravascular cooling devices. Progesterone therapy at 80-100 mg intramuscularly every 12 hours for 72 hours was initiated shortly after admission to the ICU.

We present a case series of three patients (mean age = 29.3 years, mean presenting Glasgow Coma Score = 3) who suffered ventricular fibrillation (VF) cardiac arrest from non-lightning electric shock, and who had considerably improved outcomes following the TH-progesterone combination therapy protocol. The average length of stay was 13.7 days.

The cases presented suggest that there may be a role for neuroprotective combination therapy in post-resuscitation care of VF cardiac arrest. While TH is well documented as a neuroprotective measure, progesterone administration is a safe therapy with promising, albeit currently inconclusive, neuroprotective effect. Future protocols involving TH and progesterone combination therapy in these patients should be further explored.

## Introduction

Anoxic brain injury (ABI) is the most severe consequence and the main cause of mortality following cardiac arrest [1]. ABI can cause irreversible and debilitating neurological deficits [2]. Establishing a working protocol to treat patients who are at risk for ABI after suffering a cardiac arrest is of paramount importance. Several studies have demonstrated the positive effect of therapeutic hypothermia (TH) on outcomes following ventricular fibrillation (VF) cardiac arrest [3-6]. Cooling methods differ from ice packs and cold IV solutions, to cooling vests and blankets, to endovascular heat exchange catheters. There is currently no consensus on which method is most effective. However, it is generally agreed upon that TH is considered immediately following resuscitation [3]. The literature has demonstrated that the use of progesterone in animal models has improved outcomes following cardiac arrest. A multitude of animal models has demonstrated the neuroprotective effects of therapy with progesterone and progesterone metabolites [7-10]. However, there has yet to be a consensus on its use in humans, despite several Phase II and III clinical trials [11-14]. Moreover, there has yet to be sufficient exploration of combination therapy of both progesterone and TH as a neuroprotective strategy in patients who have suffered cardiac arrest. We present a case series of three patients who suffered a cardiac arrest from electric shock who had considerably improved outcomes following a treatment protocol of TH and progesterone combination therapy.

## Case presentation

Our study consisted of a retrospective analysis of three male patients from St. Mary’s Medical Center, a Florida state-designated Level I trauma center. The average age was 29.3 years, and all three patients suffered from some form of non-lightning electric shock. The patients arrived each with a Glasgow Coma Score (GCS) of 3T with an unknown length of cardiac arrest. All patients received on-site advanced cardiac life support and a head CT scan upon arrival. They were each defibrillated from VF on-scene and arrived at the medical center with the return of spontaneous circulation (ROSC).

TH was initiated immediately upon arrival at the trauma bay. The patients were taken to the ICU and Thermaguard heat exchange catheters were placed upon arrival. On average, the duration of TH was 35.6 hours. Progesterone was administered with a dose of 80-100 mg intramuscularly every 12 hours for 72 hours. The average length of stay was 13.7 days. The patients were extubated with full neurological recovery on day 4, day 7, and day 2, respective of the order of patient presentation.

Our protocol dictates a goal temperature of 33°C with the utilization of cooling blankets, ice packs, chilled IV fluids, nasogastric lavage with iced saline, and an intravascular cooling device. To prevent shivering, patients were treated with several medications including acetaminophen, meperidine, and buspirone, or physically by using a heated gel pack, oxygen mask, or face tent. Once shivering was addressed, the patients were then sedated with either a propofol infusion starting at 5 mcg/kg/min, titrated to effect with a maximum of 50 mcg/kg/min or midazolam (initial midazolam bolus of 0.15 mg/kg to a max of 10 mg) and then with continuous midazolam (2-6 mg/hour). Furthermore, a fentanyl drip (25-75 mcg/hour) was used with either sedative agent.

 

Case 1

A 24-year-old male with no past medical history was found on the ground for an unknown period of time. The patient was trimming a high tree prior to being found next to a severed high powerline. Cardiopulmonary resuscitation (CPR) was initiated at the scene, and ROSC was achieved after 25 minutes. On arrival to the hospital, the patient was unconscious with a GCS of 3T. All the trauma workups were negative, including head CT, MRI, and electroencephalogram (EEG). Our hypothermia protocol of TH to 33°C was initiated for 18 hours, followed by progesterone 80 mg every 12 hours for 72 hours. After the conclusion of the protocol, the patient started to become more alert, oriented, and responsive to stimulations. The hospital course was complicated by ventilator-associated pneumonia, but he was extubated on hospital day 7. The patient was assessed neurologically, and he was found to have a GCS of 15 with motor and sensory abilities fully intact. Common psychoanalysis was performed immediately after extubation of two weeks, two months, and six months over the phone. The patient showed full emotional and functional recovery after hospital discharge.

 

Case 2

A 25-year-old male electrician with a history of chronic smoking arrived at the ED due to electrocution at work. The patient was immediately resuscitated with CPR by coworkers. Emergency medical services (EMS) arrived and continued CPR until they achieved ROSC after 10 minutes. The patient arrived at the hospital intubated with a GCS of 3T. On physical examination, there was an electrical burn over the left shoulder and an abrasion over the forehead. All the trauma workup were negative, including head CT, MRI, and EEG. The hypothermia protocol was immediately initiated at 33°C for 18 hours followed by progesterone 100 mg every 12 hours for 72 hours. After the hypothermia protocol, the patient was extubated on hospital day 4. The patient was assessed neurologically, and he was found to have a GCS of 15 with motor and sensory abilities fully intact. Common psychoanalysis was performed immediately after extubation, two weeks, two months, and six months over the phone. The patient showed full emotional and functional recovery after hospital discharge. 

 

Case 3

A 39-year-old male construction worker was subjected to electric shock while operating a crane and was ejected 10 feet. The patient was found in cardiac arrest, and EMS immediately started CPR. ROSC was received after 30 minutes, and he was transferred to the hospital. He was intubated with a GCS of 3T. On physical examination, he had a deep partial-thickness burn of the right hand and full-thickness burn on the left hand. He underwent a fasciotomy of his left hand. His trauma workup was remarkable for a left humerus fracture but otherwise negative. The hypothermia protocol was initiated immediately at 33°C for 18 hours followed by progesterone 80 mg every 12 hours for 72 hours. The patient tolerated the protocol well and remained intubated for two days. Afterward, he was assessed neurologically with a GCS of 15, and motor and sensory abilities were fully intact. Common psychoanalysis was performed after extubation, two weeks, two months, and six months over the phone. The patient showed full emotional and functional recovery after hospital discharge.

## Discussion

Brain injury secondary to VF cardiac arrest, particularly in patients who achieve an ROSC, is a complex, multifaceted insult to brain tissue; the initial anoxia from cardiac arrest couples with secondary brain insults such as reperfusion injury, impaired cerebral autoregulation, and fluctuations in cerebral arterial carbon dioxide, among others, after ROSC is achieved [15]. The etiologies of a brain injury range widely and include cardiac arrest, blunt force, penetrating injury, and electrocution, among others. These facets of injury convene in common, albeit complex, a pathophysiological pathway involving inflammatory cytokines, excitotoxicity, and vasogenic edema, ultimately resulting in neuronal cell death [16]. TH has been adopted as a standard in VF post-cardiac arrest care. Its use is encouraged by the American Heart Association (AHA) in all comatose patients as it is strongly associated with increased survival and neuroprotection after ROSC (AHA citation of 2015 updates). A meta-analysis of 11 studies of out-of-hospital cardiac arrest found a significant decrease in mortality and improved neurological outcomes in patients who underwent TH after ROSC [17]. Another review of six randomized controlled trials showed a 30% survival benefit for those undergoing TH therapy [18]. Animal models and clinical trials have described strong support for both TH and progesterone therapy, particularly in traumatic brain injury (TBI). TH has been consistently shown to decrease mortality and improve neurologic outcomes in TBI patients [19,20]. One systematic review demonstrated reduced mortality and enhanced neurological function after long-term hypothermia therapy in lieu of short-term therapies [20]. Additionally, cooling has successfully been used to optimize operative neurosurgical outcomes, further suggesting that TH may favorably regulate neuronal injury. Whereas the neuroprotective benefit of TH has been well established in post-cardiac arrest neuroprotection, the current discourse on neuroprotective progesterone therapy remains inconclusive. A multitude of animal models has demonstrated the neuroprotective effects of therapy with progesterone and its metabolites [7-10]. Two Phase II clinical trials have also demonstrated a significant reduction in mortality and improvements in both moderate and severe TBI. The 2007 ProTECT II study by Wright et al. demonstrated a significant reduction (rate ratio 0.43; 95% CI 0.18-0.99) in mortality compared to placebo at 30-day follow-up in both moderate and severe TBI patients after progesterone therapy [11]. A second Phase II study by Xiao et al. in 2008 observed a similar neuroprotective effect, studying patients with only severe TBI and using far lower doses of progesterone than ProTECT II [12]. Both studies noted the safety, low cost, and easy availability of progesterone as a neuroprotective drug. From these two successful Phase II studies, two large multicenter Phase III studies arose, ProTECT III and SyNAPSe. Both studies demonstrated inconsistent results with their predecessors, failing to demonstrate a neuroprotective effect in moderate and severe TBI. Speculations of why such robust findings in animal and Phase II trials were not reflected in Phase III trials have sparked further discussion on the matter, including speculations of both subtherapeutic and supratherapeutic dosing in the unsuccessful Phase III trials [7,14].

## Conclusions

Early and aggressive treatment is crucial for neurological recovery after a cardiac arrest to prevent ABI and secondary brain insults in patients with an electric shock. Although there are various methods for management, this paper focuses on TH and progesterone therapy in three different patients. Not only is it crucial to initiate the protocol after ROSC is achieved, but this combination therapy may play a neuroprotective role in post-resuscitation care of cardiac arrest. Although TH is well documented as a protective measure, progesterone administration is a safe therapy with a promising, albeit inconclusive, neuroprotective effect. The complex pathophysiology of ABI is not completely understood; however, it may be better managed through a synergistic TH and progesterone therapy rather than TH therapy alone. Unsuccessful Phase III trials testing the significance of progesterone therapy may be due to subtherapeutic and supratherapeutic dosing or the presence of other confounding factors lead to worsening of the brain injury. Future protocols involving TH and progesterone therapy in these patients should be further explored to further understand its benefit in neurological function and recovery.
